# Time trends in stroke risk management among high-risk patients with non-valvular atrial fibrillation in Australia between 2011–2019

**DOI:** 10.1016/j.ijcha.2024.101443

**Published:** 2024-06-26

**Authors:** K. Giskes, N. Lowres, J. Orchard, K. Hyun, C. Hespe, B. Freedman

**Affiliations:** a The University of Notre Dame, General Practice, Sydney, Australia; b Heart Research Institute, Sydney, Australia; c University of Sydney, Sydney, Australia

**Keywords:** Atrial fibrillation, Stroke risk reduction, non-vitamin K oral anticoagulants, Primary care

## Abstract

**Background:**

Atrial fibrillation (AF) is associated with stroke. Major changes to AF management recommendations in 2016–2018 advised that: 1. Stroke risk be estimated using the CHA_2_DS_2_-VA score; 2. Antiplatelet agents (APAs) do not effectively mitigate stroke risk; 3. Anticoagulation is prioritised above bleeding risk among high-risk patients; and 4. Non-vitamin K oral anticoagulants (NOACs) are used as first-line anticoagulants.

**Aim:**

To examine trends in stroke risk management among high-risk patients with non-valvular AF in Australia between 2011–2019.

**Method:**

De-identified data of patients were obtained from 164 separate general practices. Data included information on patient demographics, diagnoses, health risk factors and recent prescriptions. Patients with a diagnosis of non-valvular AF were identified and stroke risk was calculated by CHA_2_DS_2_-VA score. High risk patients (i.e. CHA_2_D_S_2-VA ≥ 2) were categorised as being managed by oral anticoagulants (OACs, i.e., warfarin or NOACs), APAs only, or neither (i.e., no OACs or APAs) and time trends in prescribing were examined. Multivariate analyses examined the characteristics of patients receiving the guideline recommended OAC management.

**Results:**

Data were available for 337,964 patients; 8696 (2.6 %) had AF. Most patients with AF (85.8 %, n = 7116) had high stroke risk. The proportion of high-risk patients managed on OACs increased from 56.7 % in 2011 to 73.7 % in 2019, while the proportion prescribed APAs declined from 31.1 % to 14.0 %. Those receiving neither treatment remained steady (around 12 %). Overall, 26.3 % of patients were inadequately anticoagulated at the end of the study period. There were no age or gender differences in receiving the guideline-recommended therapy, and patients with comorbidities associated with increased stroke risk were more likely to receive OAC therapy.

**Conclusions:**

Stroke risk management among patients with AF has improved between 2011–2019, however there is still scope for further gains as many high-risk patients remain inadequately anticoagulated. Better stroke risk assessment by clinicians coupled with addressing practitioner concerns about bleeding risk may improve management of high-risk patients.

## 1 Introduction

Atrial fibrillation (AF) is the most common arrhythmia and is becoming a growing health problem with the ageing Australian population. [1] If untreated, AF increases stroke risk 5-fold. [2] Around one-third of strokes are due to AF, [3] and 20 % could be prevented if stroke risk was appropriately managed. [4].

Over the past decade there have been numerous advances in AF guidelines and therapies in Australia. Since 2009 non-vitamin K antagonist oral anticoagulants (NOACs), a class of oral anticoagulants that circumvent the limitations of warfarin, are superior for stroke prevention, and associated with lower total mortality and intracranial haemorrhage, were approved in Australia, then listed on the PBS in 2013. [5] In 2016 the European Society of Cardiology (ESC) Guidelines stated antiplatelet agents (APAs) had no role in stroke risk reduction. [6] Furthermore, these guidelines recommended that anticoagulation be prioritised above bleeding risk when managing patients with high stroke risk, and that NOACs be used as first-line therapy for anticoagulation. [6] In 2018 these recommendations were adopted in the Australian 2018 CSANZ & NHF guidelines; the first Australian consensus guidelines on AF management. [2].

Australian and international research has shown increases in the proportion of patients with AF and high stroke risk being managed by oral anticoagulants (OACs). [7], [8] No Australian study has examined trends in the management of high-risk patients as defined by the current Australian guidelines (i.e., with CHA_2_DS_2_-VA score ≥ 2) or examined trends in stroke risk management for a range of anti-thrombotic agents among high-risk patients, and the characteristics of patients managed with OACs. The current study examines trends in stroke risk management among high-risk patients with AF, utilising deidentified Australian general practice data to explore these current knowledge gaps in stroke risk management.

## 2 Methods

### 2.1 Data sources

Data were obtained from baseline (pre-intervention) data from four intervention studies focussed on cardiovascular quality improvement or AF screening in general practices conducted between 2011 and 2019: the TORPEDO study (treatment of cardiovascular risk in primary care using electronic decision support, 2011–2015, n = 50806 patients), [9] AF SMART (atrial fibrillation screening, management and guideline recommended therapy, 2016–2019, n = 64863 patients), [10] QPULSE (quality improvement in cardiovascular disease outcomes in general practice, 2015–2017, n = 154972 patients) [11] and the INTEGRATE study (integrated combination therapy, electronic general practice support tool, pharmacy-led intervention and combination therapy evaluation, 2016–2019n- 67,323 patients). [12] A total of 164 practices in four states (New South Wales, Victoria, Queensland and Western Australia) were recruited by advertisements through their local Primary Health Networks and by direct approach- there was no overlap in practices between the studies. TORPEDO, INTEGRATE and AF SMART obtained approval from the University of Sydney Human Research Ethics Committee, reference numbers 13533, 2015/616 and 2017/1017, respectively. QPULSE received approval from the University of Notre Dame Australia Human Research Ethics Committee reference number UNDA 014105S. Data were accessed through a data-sharing agreement between the George Institute, the University of Sydney and the University of Notre Dame Australia.

Data were extracted from each practice using an identical method and data extraction tool (Pen CS Clinical Audit Tool. PenCAT) which ensured that all variables were derived and coded uniformly across all practices and time points. The data comprised of basic demographic information (age, sex), medical diagnoses, prescriptions, and some biometric measures (i.e., height, weight, blood pressure, blood lipids). Age (in years) was summarised as a continuous variable. Co-morbidities were available as dichotomous variables. Data were cleaned and coded as ‘missing’ when there was no recorded value/data. There was less than 5 % missing data for each of the variables examined.

### 2.2 Eligible patients

Patients were eligible if they met the following criteria: age ≥ 18 years; diagnosis of non-valvular AF; and an ‘active patient’ at the practice (i.e., had attended the practice at least three times in the previous 24 months, and at least once in the previous 6 months [13]).

### 2.3 High stroke risk assessment

CHA_2_DS_2_-VA score ≥ 2 was used to identify high-risk patients. CHA_2_DS_2_-VA co-morbidities were determined from coded variables as outlined in Box 1. The CHA_2_DS_2_-VA score (range 0–9 points) is the sum of: congestive heart failure/left ventricular dysfunction (1 point); high blood pressure (1 point); age > 75 years (2 points); diabetes (1 point); stroke/transient ischaemic attack/thromboembolism (2 points); vascular disease (coronary artery disease, myocardial infarction, peripheral artery disease, aortic plaque) (1 point); age 65–74 years (1 point). The current guidelines advocate oral anticoagulation (OAC) among this group and recommend a NOAC as first-line agent, unless contraindicated. [2].

Box 1 **.**

**Table:** 

**Co-morbidity**	**Coded diagnosis**
Congestive heart failure	‘heart failure’, ‘congestive heart failure’, ‘right-/left-/biventricular heart failure’, or ‘cor pulmonale’
Hypertension	‘hypertension’, average systolic blood pressure > 140 mmHg or average diastolic blood pressure > 90 mmHg across the last three blood pressure readings, or a current prescription for one or more anti-hypertensive agents
Diabetes	‘diabetes’ (excluding gestational diabetes)
Stroke	‘stroke’ or’transient ischaemic attack’
Vascular disease	‘peripheral vascular disease’ or ‘myocardial infarct’

### 2.4 Stroke risk management

Prescription data for the previous six months were used to ascertain ‘current’ stroke risk-reduction management. Management was categorised as either having a current script for an OAC, APA only or neither (i.e., no APA and no OAC). OACs included warfarin, apixaban, dabigatran or rivaroxaban; this group was further categorised into warfarin and NOACs (apixaban, dabigatran or rivaroxaban). The ‘APA only’ group included patients prescribed aspirin, clopidogrel, dipyridamole, prasugrel or ticagrelor, who had no current prescription for OACs. In cases where patients received a script for both warfarin and a NOAC in the previous 6 months, the most recent script issued was assumed to be their current treatment.

### 2.5 Analyses

Sample characteristics were presented as frequencies and percentages for categorical data and means and standard deviation (SD) for continuous variables. The proportion of in-scope patients in each management group was graphed for each time point between 2011–2019. The Cochran-Armitage test was used was used to test for time trends.

Multilevel logistic regression models were used to examine OAC prescribing by a range of patient and co-morbidity characteristics. The models were adjusted for age, sex, congestive heart failure, hypertension, diabetes, peripheral vascular disease and stroke, and accounted for the clustering effect for the individual studies. Further analyses examined the demographic and health characteristics of the high-risk patients that had a current script for an OAC in the later part of the study period (2016–2019).

All analyses were performed using SAS 9.4 (SAS Institute Inc., Cary, NC, USA). Variables in the models described above that were p <= 0.05 (two-tailed) were considered significant.

## 3 Results

**Fig. 1:**
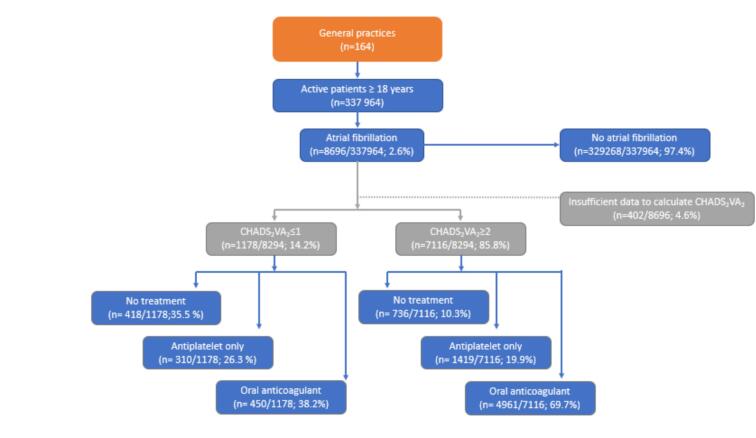
Study sample flow chart.

Across the 164 practices, there were data from 337,964 patients aged ≥ 18 years. Of these, 2.6 % (n = 8696) were diagnosed with non-valvular AF. Sufficient data for calculation of CHA_2_DS_2_-VA score were available for 95.4 % (n = 8294). Most patients with AF had high stroke risk (CHA_2_DS_2_-VA ≥ 2; 85.8 %, n = 7116), and 69.7 % (n = 4961) had a current script for OACs; 19.9 % (n = 1419) received APAs only; and 10.3 % (n = 736) did not have a current script for either treatment (Fig. 1).

The characteristics of patients with high stroke risk over the entire study period are summarised in Table 1. As would be expected, OAC prescription was associated each of the variables comprising the CHA_2_DS_2_-VA score, as each of these variables are associated with increased stroke risk. Table 1 shows that more than 70 % of patients with co-morbidities related to increased stroke risk (congestive heart failure, hypertension, diabetes, vascular disease and previous stroke) were prescribed an OAC. Just over half (52 %, n = 3728) were male, and most had hypertension. Around one-quarter had diabetes (27 %, n = 1907) and 15 % (n = 1060) had a prior stroke. This was confirmed in the multivariate analyses, which showed no gender differences in being prescribed the guideline-recommended treatment (CHA_2_DS_2_-VA score is sexless), and patients with co-morbidities related to increased stroke risk were more likely to be managed with an OAC. The only exception to this was patients with vascular disease. There were no age differences in OAC prescribing among high-risk patients.

**Table 1:** Characteristics of in-scope patients all years combined.

	**Total (all years combined)**				**Adjusted multilevel logistic model for management with OAC (all years combined)**		
	Total (%) N = 7116	OAC N = 4961	No OAC N = 2155	p-value	Variable	OR (95 % CI)	P-value
**N = 7116**							
Age (mean, SD)	78 (10)	78 (9)	78 (11)	0.5728	Age	1 (1.00––1.01)	0.5693
Males (%)	3728 (52 %)	2632 (71 %)	1096 (29 %)	0.0940	Sex (female)	1.08 (0.97––1.20)	0.1590
**Comorbidities**							
Congestive heart failure (%)	1171 (16 %)	857 (73 %)	314 (27 %)	0.0047	Congestive heart failure	1.27 (1.09––1.49)	0.0028
Hypertension (%)	6687 (94 %)	4717 (71 %)	1970 (29 %)	0<.0001	Hypertension	1.90 (1.55, 2.33)	0<.0001
Diabetes (%)	1907 (27 %)	1381 (72 %)	526 (29 %)	0.0027	Diabetes	1.22 (1.08, 1.37)	0.0016
Vascular disease (%)	274 (4 %)	193 (70 %)	81 (30 %)	0.7909	Vascular disease	0.95 (0.72, 1.24)	0.6894
Stroke (%)	1060 (15 %)	820 (77 %)	240 (23 %)	0<.0001	Stroke	1.63 (1.39, 1.9)	0<.0001

**Fig. 2:**
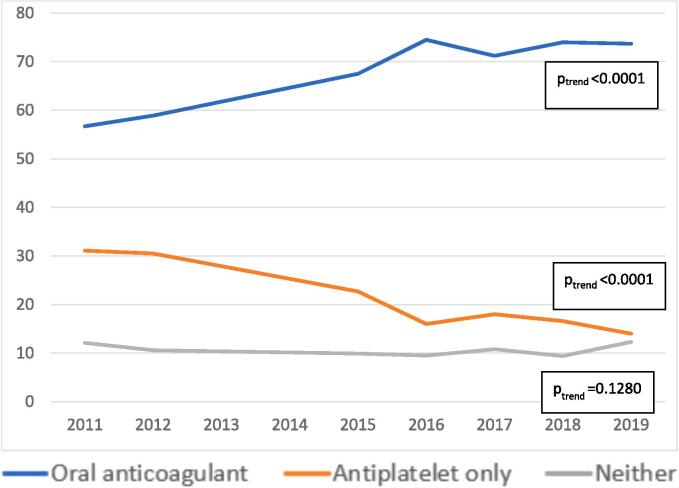
Proportion of patients with atrial fibrillation and high stroke risk with a current script for oral anticoagulants, antiplatelet agents or neither treatment between 2011 to 2019. Fig. 2 legend.

Time trends in management of high-risk patients are shown in Fig. 2. Over the study period there was a significant increase in those managed on OACs from 56.7 % in 2011 to 73.7 % in 2019 (p for trend < 0.0001). The trend persisted even after adjusting for patient characteristics (adjusted odds ratio (aOR): 1.12, 95 % confidence interval (CI): 1.07, 1.17). There was a corresponding decline in patients receiving APAs only from 31.1 % in 2011 to 14.0 % in 2019 (p for trend < 0.0001). The adjusted multilevel model also showed a similar negative trend of APA only use over time (aOR: 0.88, 85 % CI: 0.85, 0.90) The proportion of patients with no current script for OACs or APAs remained consistent around 12 % over the study period (p for trend = 0.128, aOR: 0.97, 95 % CI: 0.92, 1.01).

**Fig. 3:**
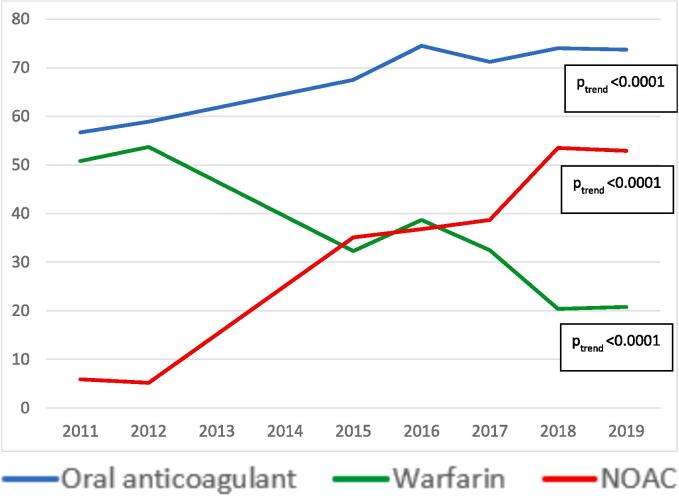
Proportion of patients with atrial fibrillation and high stroke risk with a current script for oral anticoagulants (combined) and warfarin and NOACs between 2011 to 2019. Fig. 3 legend.

The percentage of high-risk patients managed on warfarin declined from 50.8 % in 2011 to 20.8 % in 2019 (p for trend < 0.0001), which also persisted after adjustments (aOR: 0.91, 95 % CI: 0.85, 0.98). During this time there was a sharp up-take of NOACS, from 5.9 % in 2011 rising to over half (52.9 %) in 2019 (p for trend < 0.0001, aOR: 1.2, 95 % CI: 1.12, 1.28).

## 4 Discussion

This large study showed marked improvements in stroke risk management of patients with AF in Australia between 2011–2019, with an increasing proportion of patients receiving the guideline- recommended OAC treatment. This was mirrored by a sharp decline in those receiving APAs alone. Despite these promising trends, at the end of the study period a large proportion of high-risk patients were not adequately anticoagulated, and improved management among these patients may lead to larger reductions in avoidable strokes. Better practitioner assessment of patient’s stroke risk, as well as addressing barriers about bleeding risk may improve stroke risk management among this group.

The improvements in management seen in the current study are in line with other Australian and international studies using general practice [8], hospital registry [14] and market data. [5] A combined-country analysis of 35 countries of n = 51 270 patients with newly diagnosed AF and at least one stroke risk factor showed that between 2010 and 2016 the proportion commenced on an OAC increased from 42.1 % to 57.7 %. [15] A recent Australian study using National Prescribing Service (NPS) data showed a rise in OAC prescribing of similar magnitude (approximately 15 %) over the same period, [8] which is comparable to the 17 % rise in OAC management seen in the current study. No known Australian study has documented trends in OAC prescribing for AF after 2019. Studies in Australia and internationally have also shown a reduction in warfarin and corresponding increase in NOAC use over this period. [5], [8] Similar to the current study, the use of APAs as monotherapy for patients who have AF with increased stroke risk has also declined: in the combined countries analyses this reduced from 30.2 % to 16.3 % between 2010 and 2016. [15] These trends may be due to promotion and awareness of the 2016 ESC and 2018 Australian management guidelines as well as the marketing of NOACs and their inclusion on the PBS.

The results showed that neither patients’ age nor gender was associated with being managed by an OAC. Previous studies have shown that females [16] and older patients [17] with AF are less likely to receive adequate anticoagulation. Changes to therapies available and the guidelines over the period covered by this study may have contributed to these findings. Bleeding risk is a major barrier reported by practitioners for initiating anticoagulation among patients with AF, [16] particularly among older patients. [17].

We had a limited number of variables extracted from the electronic medical records and did not have any data pertaining to bleeding risk or history of bleeding available from the data extracts. Furthermore, these data sets did not contain information about contraindications to OAC prescription. The introduction of NOACs which have a lower risk of major bleeding, particularly intracranial haemorrhage, and require less monitoring than warfarin, may have reduced some barriers to practitioners prescribing anticoagulation. [18] However, bleeding remains a risk when taking NOACs and this may still be a barrier to practitioners prescribing them. We also did not have data on the use of left atrial appendage (LAA) closure devices to reduce stroke risk among these high-risk patients in this study. However, LAA devices are rarely used in Australia- thoroscopic LAA device placement was first approved under the Medicare Benefits Scheme (MBS) in 2017, with only 160 devices implanted in the entire country in 2017, rising to 460 in 2019/20 (end of the study period) [18].

Further gains may be achieved by targeting GPs’ knowledge of the new guideline recommendations, particularly in relation to bleeding risk, and increasing their confidence to prescribe OACs (and NOACs in particular) to patients they may have previously considered unsuitable for anticoagulation. The results also showed that patients with co-morbidities increasing stroke risk were more likely to be managed with an OAC, which suggests that clinicians are aware of some of these increased risks but either may not be aware of the formal assessments of stroke risk, and/or may not been reviewing patient’s stroke risk regularly.

Although the trends seen in the current study were similar to other studies, the estimates of the proportion of patients receiving OACs was slightly higher in the current study compared to those previously reported in other Australian studies. The proportion of high-risk patients managed with an OAC toward the end of the (2019) (73.7 %) was higher than reported in an Australian general practice study in 2018 (55.2 %) [8] and another study reporting 63 % based on hospital admission data. [14] The higher rates in the current study may be due to geographic location and participation bias: most practices (80.5 %, n = 132) in the current study were in the Greater Sydney area and consented to participate in a research study. The studies also differed in their measurement of stroke risk. One large Australian study reporting a lower proportion of OAC prescription (55 %), calculated high stroke risk using CHA_2_DS_2_-VASc ≥ 2 for both females and males [8], which is not aligned with the current guidelines. Therefore their sample denominator would have included females with intermediate risk who did not have a guideline OAC recommendation, as the guidelines categorise females with CHA_2_DS_2_-VASc ≥ 2 as intermediate risk and females with CHA_2_DS_2_-VASc ≥ 3 as high risk. The trends in OAC and warfarin use for patients with AF and high stroke risk mirror those trends documented internationally in several Asian countries, Europe, the USA and UK [7].

### 4.1 Strengths and limitations

There are several limitations to that need to be considered in context of the study. Most participating practices were in Greater Sydney; therefore, the results have limited generalisability to rural areas and other states. The data only contained prescriptions issued by general practitioners, therefore patients who received their scripts from their cardiologist may have been incorrectly identified as receiving no treatment. Furthermore, aspirin is available without a prescription, which may have under-estimated the proportion of patients managed on an APA alone but is unlikely to affect the trend of declining APA use. This study used data extracted from a limited number of fixed fields of the medical records- therefore information such as co-morbidities/bleeding risk and contraindications to OAC prescription entered in the electronic medical record may not have been captured in the current data- which may have led to an under-estimation of AF diagnosis and stroke risk but is unlikely to have affected the trends observed.

## 5 Conclusion

There have been marked improvements in the past 10 years in the proportion of high-risk patients receiving appropriate stroke-reducing therapies in line with guideline recommendations. This is likely to have a salutary effect on reducing AF-related stroke, Further reductions in stroke could be achieved by targeting the approximately 25 % of patients at high stroke risk who do not receive the recommended OAC treatment: about half of these patients are receiving inadequate anticoagulation by management with APAs alone, and the remainder receive no antithrombotic therapy. Addressing practitioner stroke risk assessment and barriers to OAC use may further improve management.

## CRediT authorship contribution statement

**K. Giskes:** Writing – review & editing, Writing – original draft, Methodology, Formal analysis, Data curation, Conceptualization. **N. Lowres:** Writing – original draft, Supervision, Formal analysis, Data curation, Conceptualization. **J. Orchard:** . **K. Hyun:** Formal analysis, Data curation. **C. Hespe:** Writing – review & editing, Writing – original draft, Supervision, Methodology, Data curation, Conceptualization. **B. Freedman:** Writing – review & editing, Writing – original draft, Supervision, Methodology, Formal analysis, Data curation, Conceptualization.

## Declaration of competing interest

The authors declare that they have no known competing financial interests or personal relationships that could have appeared to influence the work reported in this paper.
